# Harnessing CRISPR technology for viral therapeutics and vaccines: from preclinical studies to clinical applications

**DOI:** 10.1016/j.virusres.2024.199314

**Published:** 2024-01-12

**Authors:** Farzaneh Zahedipour, Fatemeh Zahedipour, Parvin Zamani, Mahmoud Reza Jaafari, Amirhossein Sahebkar

**Affiliations:** a Microbiology Department, Medical Sciences Branch, Islamic Azad University (IAU), Tehran, Iran; b Nanotechnology Research Center, Pharmaceutical Technology Institute, Mashhad University of Medical Sciences, Mashhad, Iran; c Department of Medical Biotechnology and Nanotechnology, Faculty of Medicine, Mashhad University of Medical Sciences, Mashhad, Iran; d Department of Pharmaceutical Nanotechnology, School of Pharmacy, Mashhad University of Medical Sciences, Mashhad, Iran; e Biotechnology Research Center, Pharmaceutical Technology Institute, Mashhad University of Medical Sciences, Mashhad, Iran; f Applied Biomedical Research Center, Mashhad University of Medical Sciences, Mashhad, Iran

**Keywords:** CRISPR, Viruses, Treatment, Vaccine, Gene editing

## Abstract

The CRISPR/Cas system, identified as a type of bacterial adaptive immune system, have attracted significant attention due to its remarkable ability to precisely detect and eliminate foreign genetic material and nucleic acids. Expanding upon these inherent capabilities, recent investigations have unveiled the potential of reprogrammed CRISPR/Cas 9, 12, and 13 systems for treating viral infections associated with human diseases, specifically targeting DNA and RNA viruses, respectively. Of particular interest is the RNA virus responsible for the recent global outbreak of coronavirus disease 2019 (COVID-19), which presents a substantial public health risk, coupled with limited efficacy of current prophylactic and therapeutic techniques. In this regard, the utilization of CRISPR/Cas technology offers a promising gene editing approach to overcome the limitations of conventional methods in managing viral infections. This comprehensive review provides an overview of the latest CRISPR/Cas-based therapeutic and vaccine strategies employed to combat human viral infections. Additionally, we discuss significant challenges and offer insights into the future prospects of this cutting-edge gene editing technology.

## Highlights

• Recent investigations have unveiled the potential of reprogrammed CRISPR/Cas9 and CRISPR/Cas13 systems for treating viral infections associated with human diseases, specifically targeting DNA and RNA viruses, respectively.
• Of particular interest is the RNA virus responsible for the global outbreak of coronavirus disease 2019.
• This comprehensive review provides an overview of the latest therapeutic and vaccine strategies employed to combat viral diseases in humans using CRISPR/Cas systems.
• It explores significant challenges, discusses potential applications, and offers valuable insights into the future prospects of this cutting-edge gene editing technology.

## 1 Introduction

Viral infections pose significant threats to global health, and despite extensive efforts to combat them, progress has been limited. Persistent viral infections result from a combination of factors, including the emergence of antiviral resistance mutants and the development of long-term infections leading to chronic diseases. Novel approaches are imperative to eliminate even residual viruses within the host. (Bayat et al., 2018) In this regard, the CRISPR/Cas technology has emerged as a natural system uniquely poised to address viral infections comprehensively. Moreover, this technology has evolved into a powerful toolbox applicable across diverse detection fields. (Cheng et al., 2022; Ma et al., 2023)

**Fig. 1:**
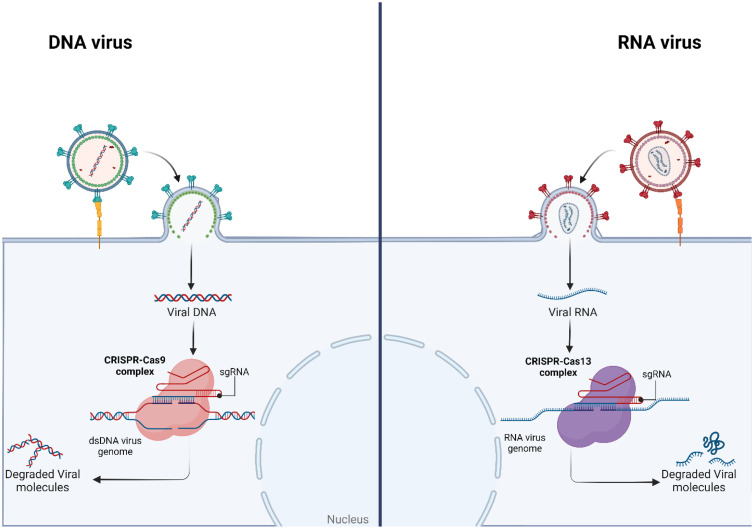
The Application of CRISPR/Cas Systems for Antiviral Therapy.

In terms of treatment strategies, CRISPR/Cas-based interventions hold promise for both acute and chronic infections, with particular efficacy demonstrated in the latter. Viral latency, a stage limiting viral activity, is a primary contributor to the frequent development of clinically important viral infections into chronic conditions. Current treatments often prove ineffective in these situations. Concurrently, CRISPR/Cas technology facilitates the identification of new antiviral candidates and the development of innovative disease models. A notable advantage of the CRISPR/Cas system lies in its adaptability. While the Cas9 protein has garnered extensive attention, Cas12 and Cas13 exhibit remarkable antiviral potential. (Li et al., 2020) Given that the genetic material of viruses can be either RNA or DNA, this is a topic of major attention in antiviral therapy. DNA viruses with dsDNA genome can be effectively targeted and inhibited by Cas12a and Cas9. Contrarily, Cas13 may specifically target negative- and positive-sense RNA viruses (Fig. 1). Additionally, by targeting RNA, Cas13 cannot result in permanent genetic changes in host cells, thus it is a safer option for in vivo applications. (Li et al., 2020) It is interesting to note that researchers have also looked into Cas13′s potential as a prophylactic treatment for SARS-CoV-2 infection. These nucleases could well be coupled with antiviral drugs and other biomolecules, considerably extending therapeutic options as well as the inherent diversity of the CRISPR/Cas system. (TR Abbott et al., 2020)

In addition, a Cas9 orthologue known as Francisella novicida Cas9 (FnCas9) exhibits the unique capability to target RNA. This distinct feature has been harnessed for the targeting of several human positive-sense single-stranded RNA viruses, including the human hepatitis C virus (HCV), tobacco mosaic virus (TMV), and the cucumber mosaic virus (CMV). (Sharma et al., 2021) The utilization of FnCas9 has shown varying levels of effectiveness against these viruses, showcasing its potential as a versatile tool in combating RNA-based viral infections. This novel approach expands the scope of CRISPR technology, offering a promising avenue for addressing a broader spectrum of viral threats beyond traditional DNA-targeting mechanisms. (Zhang et al., 2018)

Theoretically, the targeted cleavage of the virus's genome within the host could render it inactive, thereby reducing or terminating the viral infection. This adaptability feature of CRISPR/Cas technology makes it as a versatile and potent tool with far-reaching implications for the treatment and understanding of viral infections.

This comprehensive review outlines the recent advancements in utilizing CRISPR/Cas systems, specifically focusing on Cas9, Cas13, and Cas13, for addressing pathogenic human viruses with both DNA and RNA genomes. We further, go on to discuss the novel CRISPR/Cas approaches that has recently been developed to tackle SARS-CoV-2 infection and can be utilized for other RNA viral infections. In addition, we comprehensively review recent vaccine platforms developing by CRISPR technology. Finally, we discuss some challenges, potential solutions, and future perspective of this cutting-edge technology.

## 2 CRISPR/Cas and DNA viruses

### 2.1 Hepatitis B virus (HBV)

Hepatitis B infection is significant risk factor for developing liver diseases and liver cancer. (Sharma et al., 2021) The HBV DNA genome, which adopts a circular structure, consists of a concise sense strand and a lengthier anti-sense strand, rendering it partially double-stranded. The pivotal element in sustaining HBV viability and contributing to the challenges in eradicating the virus lies in the episomal covalently closed circular DNA (cccDNA) structure of the HBV genome. (Dandri and Petersen, 2016) Efforts to treat HBV involve the inactivation of HBV cccDNA, a challenging task given its location within the nucleus. As a potential anti-HBV therapy, gene editing can be utilized in tandem with gene-silencing technology to address this challenge. (Zhu et al., 2019) The simultaneous cleavage of several HBV genome regions using CRISPR/Cas9 with multiplexed guide RNAs (gRNAs) has proven to augment the effectiveness of HBV genome ablation and elimination. (Sakuma et al., 2016) Additionally, some studies asserted that the HBV integrated cccDNAs might be destroyed or rendered inactive by the CRISPR/Cas9 system. (H Li et al., 2017) Researchers have achieved the disruption of HBV cccDNA in a stable HBV cell line, utilizing CRISPR/Cas9 technology to entirely delete a full-length 3175 bp HBV DNA from the host genome. (H Li et al., 2017) As a result, gene editing holds significant potential strategy for limiting HBV infection.

In both *de novo* and chronic in vitro cell cultures of HBV, the application of SpCas9, along with sgRNAs directed towards the HBV core protein, reverse transcriptase (RT), or surface antigens, effectively facilitated the degradation of HBV DNA, leading to a reduction in cccDNA levels. (Kennedy et al., 2015) Targeting of HBV by CRISPR-Cas9 has been successfully tested in liver cell lines HepG2, Huh-7, HepG2-H1.3, HepG2.A64 and HepG2.2.15. (H Li et al., 2017; Karimova et al., 2015; Lin et al., 2014; Zhen et al., 2015) This targeting caused cccDNA to be disrupted and viral replication to be reduced. Liu et al. report that SpCas9 and eight gRNAs can be bind the conserved areas of various HBV genotypes and led to the reduction of HBV replication both in vitro and in vivo. (Liu et al., 2015) Even though these experiments have demonstrated the potential of CRISPR/Cas9 in the eradication of HBV infection, additional research is required to enhance our knowledge of HBV replication and cccDNA biology to create efficient editing systems for eliminating the HBV infection.

### 2.2 Epstein-Barr virus (EBV)

EBV is among the most commonly widespread infection that affects humans. Although EBV infection is typically asymptomatic, it can occasionally result in serious diseases such as B-cell lymphomas, NK/T-cell lymphomas, and EBV-related lymphoproliferative disease. (Kieff, 2007) Moreover, extensive research has been conducted to explore the associations between Epstein-Barr virus (EBV) infection and malignancies originating from epithelial cells, such as gastric and nasopharyngeal carcinomas. (Kanda et al., 2016)

The CRISPR/Cas9 system has been utilized to target herpesviruses, particularly EBV, and some EBV-associated diseases caused by its latency. In order to achieve various editing goals, Wang et al. designed seven guide RNAs that targeted six distinct areas in the genome of EBV. They transfected these sgRNAs to a B cell line obtained from Burkitt's lymphoma patients. The CRISPR-SpCas9/sgRNAs resulted in a reduction of the episomal EBV genome within infected cells. This intervention led to a substantial arrest in cell growth and a corresponding decline in viral load. (Wang and Quake, 2014) Later, Yuan et al. designed two sgRNAs that target a 558 bp area in the BART (*Bam*HI A rightward transcript) promoter region. This region is a significant viral transcript that is synthesized by cells with the latent infection that encodes the viral microRNA. In nasopharyngeal cancer cells exhibiting latent EBV infection, the identification of microRNA gene disruption hinted at a potential therapeutic avenue for addressing EBV infection. (KS Yuen et al., 2015) Moreover, it has been documented that in Burkitt's lymphoma Akata-Bx1 cells, the use of a sgRNA directed towards the Epstein-Barr virus nuclear antigen 1 (EBNA1) and the origin of replication (ori) region resulted in the disruption of a substantial portion of the EBV genome, approximately 40–60 %. Simultaneously targeting EBNA1 with two distinct sgRNAs led to the disruption of a significant segment of the gene, exceeding 90 %. (van Diemen et al., 2016)

The entry of Epstein-Barr virus (EBV) into human cells is facilitated through the involvement of Ephrin receptor tyrosine kinase A2 (EphA2). Experimental CRISPR/Cas9-mediated deletions demonstrated that the extracellular domain of EphA2 can interact with the EBV glycoprotein gHgL, thereby aiding in the facilitation of cell entry. (J Chen et al., 2018) Therefore, EphA2 may represent a novel potential therapeutic target that needs to be studied more.

### 2.3 Human papillomavirus (HPV)

Nearly all cases of cervical carcinoma and a growing number of cases of anal and head and neck cancer are caused by HPVs. (Hsu et al., 2018) HPV16 and HPV18, known as high-risk human papillomaviruses (HR-HPVs) are thought to be a major cause of cervical cancer. Oncogenes E6 and E7 are generated in the initial phases of HPV infection, and have the ability for the destruction of the normal cell cycle and sustain tumorigenesis. (Hu et al., 2014) Cas9 nuclease have been used successfully to target the E6 or E7 oncogenes in HPV-induced cancer cell lines to cleave the HPV genome leading to the elevation of p53 or pRb expression and induction of cancer cell death. (Kennedy et al., 2014) In a study conducted by Zhen et al., promising outcomes were observed in the treatment of cervical cancer through the combination of the anti-cancer drug cisplatin with Cas9. The objective was to target both E6 and E7, both in vitro and in vivo settings. (Zhen et al., 2016)

Yoshiba et al. emphasized the potential of CRISPR/Cas9 as a specialized alternative therapy for cervical cancer. In animal models exhibiting high-risk HPV-positive cervical cancer, the transduction of adeno-associated viruses (AAVs) carrying Cas9/sgRNA targeting E6 led to the inhibition of tumor cell proliferation following intratumoral administration in a mouse model with sgRNAs directed towards the E6 gene. (T Yoshiba et al., 2019) Kennedy et al. developed 18 distinct guide RNAs (sgRNAs) tailored to selectively target the integrated open reading frames (ORFs) of E6 and E7 in vitro. The results showed that, the CRISPR/SpCas9 in SiHa cells transformed with HPV-18 and HeLa cells transformed with HPV-16, successfully facilitated the precise cleavage and inhibition of the E6 and E7 genes, respectively. (Kennedy et al., 2014; S Chen et al., 2018)

### 2.4 Herpes simplex virus (HSV)

The Alphaherpesvirinae subfamily of the Herpesviridae includes two important human viruses, HSV-1 and HSV-2. (Finnen and Banfield, 2018). Herpes simplex keratitis and cold sores, leading causes of corneal blindness, are induced by HSV-1, while HSV-2 is accountable for genital herpes. (Hill et al., 2014) While the precise mechanism of its action remains elusive, the HSV-1 tegument protein UL7 is a highly conserved factor implicated in proliferation and viral infections. Notably, the CRISPR/Cas9-engineered UL7 mutant (UL7-MU strain) exhibited a tenfold decrease in replication compared to the wild-type strain. Mice infected with the UL7-MU strain demonstrated a diminished pathogenic response relative to those infected with the wild-type HSV-1 strain. Additionally, during latency, animals infected with UL7-MU displayed reduced levels of viral latency-associated transcript (LAT) expression in the brain and trigeminal nerve compared to mice infected with wild-type HSV-1. (Xu et al., 2016) This highlights the pivotal role of the HSV-1 tegument protein UL7 in viral proliferation and pathogenesis, shedding light on potential avenues for therapeutic interventions.

### 2.5 Human cytomegalovirus (HCMV)

The beta-herpesvirus, HCMV, establishes a lifelong infection in individuals, frequently remaining asymptomatic in healthy hosts but posing the potential for severe or fatal diseases in immunocompromised individuals. Given HCMV's exclusive ability to propagate within human cells, it has developed defensive mechanisms to counteract host responses to stress and manipulate cellular functions, ensuring the sustenance of its life cycle. (Y-C Chen et al., 2018) The activation of the mammalian target of rapamycin complex 1 (mTORC1) is a critical aspect of HCMV's effective infection, regulating cell proliferation and anabolic metabolism. Inhibitors of mTORC1 have proven effective in reducing the likelihood of HCMV reactivation in transplant recipients and inhibiting HCMV replication in vitro. Additionally, the pivotal HCMV protein, pUL38, plays a crucial role in averting cell death induced by endoplasmic reticulum stress during infection and can stimulate mTORC1 through interaction with and inhibition of tuberous sclerosis complex protein 2 (TSC2). Successful application of CRISPR/Cas9 technology in generating TSC2 knockout U373-MG cells underscores that pUL38 can activate mTORC1 via both TSC2-dependent and -independent mechanisms. (Bai et al., 2015) These innovative findings point to the development of complex mechanisms by which mTORC1 is activated by HCMV, highlighting the role of mTORC1 in the biology and pathophysiology of HCMV.

### 2.6 Human herpesvirus 8 (HHV-8) or kaposi's sarcoma herpesvirus (KSHV)

HHV-8 is the cause of Kaposi's sarcoma (KS), a highly vascular tumor of lymphatic and blood vessels. For KSHV gene expression and virion formation, KSHV protein ORF45 activates the cellular kinase RSK (p90 ribosomal S6 kinase) which is a main functional mediator of ERK/MAPK signaling pathway. Additionally, it has been demonstrated that ORF45 helps to maintain ERK and RSK activation throughout KSHV lytic replication. (van Diemen and Lebbink, 2017) In the study conducted by Avey et al., the CRISPR/Cas9 technique was employed to eliminate RSK, confirming its essential role in facilitating efficient lytic replication of KSHV. In this study, the importance of many downstream substrates for ORF45/RSK-dependent transactivation and KSHV progeny virion generation was clarified. This research demonstrated a critical function for the ORF45/RSK/eIF4B signaling axis in the mRNA translation with highly structured 5′-untranslated regions (UTRs), which has significant indications for KSHV pathobiology and can be targeted for the treatment of this infection. (Avey et al., 2015) Table 1 contain preclinical investigations on CRISPR/Cas for the treatment of DNA virus infections.

**Table 1:** CRISPR/Cas research for the treatment of different DNA virus infections.

Type of infection	Disease-Related Gene/Protein	Results	References
HBV	S, X, C, P	Down-regulation of HBsAg^f^, HBeAg e, HBV DNA, and HBV RNA	. (Jiang et al., 2017)
HBV	Repeated core region	Down-regulation of HBsAg, HBeAg, HBV DNA, cccDNA b with no off-target effects	. (H Li et al., 2017)
HBV	NTCP h	Disruption of NTCP expression and inhibition of the entry of HBV	. (Lin et al., 2015)
HBV	HBsAg and HBeAg	Inhibition of both HBeAg and HBsAg expression	. (H Li et al., 2018)
HBV	S, P, C	Down-regulation of HBsAg, HBeAg, HBV DNA, pgRNA, cccDNA, rcccDNA with no off-target effects	. (Y Liu et al., 2018)
HBV	RT i, P1, XCp	Down-regulation of HBsAg, HBV DNA, cccDNA, HBV RNA with no off-target effects	. (Schiwon et al., 2018)
HBV	PreS1, S2, S	Down-regulation of HBsAg, decreased the Proliferation and tumorigenicity, IL-6, and pSTAT3 in host cells.	. (Song et al., 2018)
EBV	BART a	EBV gene disruption in infected nasopharyngeal carcinoma cells.	. (S Chen et al., 2018)
EBV	EphA2 d	Reduced the expression of EphA2 on the cell surface to that of the negative control. Reduced EBV fusion and infection in cells lacking EphA2.	. (J Chen et al., 2018)
EBV	EBNA1 c	Depletion of 40–60 % of EBV genome in Burkitt's lymphoma Akata-Bx1 cells.	. (van Diemen et al., 2016)
EBV	pBART	Loss of miR-BART expression and activity due to deletion of the whole pBART locus.	. (K-S Yuen et al., 2015)
HPV	E7	Restored the production of pRb protein, enhanced the apoptosis in cells, and suppressed the proliferation in HPV16 positive cervical cancer SiHa and Caski cell lines.	. (Hu et al., 2014)
HPV	E6	Decreased E6 expression was accompanied by elevated p53 expression, apoptosis, and cellular proliferation.	. (T Yoshiba et al., 2019)
HSV	UL7	Reduced the transcription of the immediate early gene α−4, reduced neuro-virulence, pathogenic impact, expression of the latency-associated viral transcript, and decreased the genome replication.	. (Xu et al., 2016)
HSV	ICP0 g	The disintegration of promonocytic leukemia nuclear bodies caused by HSV-1 was reversed when ICP0 was disrupted.	. (Roehm et al., 2016)
HCMV	TSC2	Development of the TSC2 knockout U373-MG strain.	. (Bai et al., 2015)
KSHV	RSK1/2	Reduction of virion generation, late lytic gene expression, and RSK substrate phosphorylation.	. (Avey et al., 2015)

a BART; *Bam*HI A rightward transcript)

b cccDNA; covalently closed circular DNA

c EBNA1; EBV nuclear antigen 1.

d EphA2; ephrin receptor tyrosine kinase A2.

e HBeAg; hepatitis B e antigen.

f HBsAg; hepatitis B surface antigen.

g ICP0; Infected Cell Polypeptide 0.

h NTCP; sodium-dependent uptake transporter.

i RT; reverse transcriptase.

## 3 CRISPR/Cas and RNA viruses

### 3.1 HIV

Acquired immunodeficiency syndrome (AIDS) is a condition caused by HIV infection, leading to the deterioration of the human immune system. (Shah et al., 2014) HIV exists in two distinct variants: HIV-1 and HIV-2. HIV-2 is characterized by lower virulence and reduced transmissibility compared to HIV-1. Therefore, the primary contributor to AIDS, HIV-1 is now the focus of efforts to prevent and treat the disease. (Zhu et al., 2019) Although highly active antiretroviral therapy has rendered AIDS a manageable condition, the sustained, long-term management of AIDS poses a significant challenge. Unfortunately, latent viral reservoirs persist despite the effectiveness of highly active antiretroviral treatment, maintaining HIV-1/AIDS as a chronic and incurable infection. Moreover, in the management of HIV-1/AIDS, considerations should encompass drug resistance, potential adverse effects, and the substantial costs associated with therapy. Therefore, there is an urgent need for innovative treatment approaches to halt HIV-1 replication and eradicate latent reservoirs. (Zhu et al., 2019)

Identical repeated DNA sequences known as long terminal repeats (LTRs) play a crucial role in facilitating the integration of retroviral DNA into the host chromosome, and their presence has been noted to initiate HIV-1 gene expression. The transcription of the virus driven by LTRs may undergo alterations due to genetic variations in the LTR binding sites. (Shah et al., 2014) Both the 5′ and 3′ ends of the genome of proviruses include these elements. Furthermore, the majority of the HIV-1 genome as well as all viral protein-encoding sequences may be completely deleted if both LTRs are simultaneously cut off and the chromosomal termini are then joined. (G Wang et al., 2018) By focusing on HIV-1 LTR, Ebina et al. were able to effectively employ CRISPR/Cas9 to reduce the expression of HIV-1 genes in Jurkat cell lines. As a consequence, the transcription and replication of the HIV-1 provirus were effectively inhibited. More significantly, it demonstrated that CRISPR/Cas9 could remove internal integrated viral genes from the genome of the infected host cell, indicating that it may be a strategy for HIV-1/AIDS therapy. (H Ebina et al., 2013)

Liao et al. propose that the efficacy of eliminating and cleaving non-integrated genome of proviruses can be enhanced by targeting multiple loci within the HIV-1 genome. . (H Liao et al., 2015) They showed that combining two powerful sgRNAs that target various HIV genome areas can block viral proliferation and escape. (Lebbink et al., 2017) Wang et al. also showed that lentiviral vector delivery of *Staphylococcus aureus* Cas9 (SaCas9)/gRNAs could remove latent HIV-1 provirus and prevent provirus reactivation. It has also been demonstrated that the mutational inactivation of the HIV-1 provirus can be performed by CRISPR/Cas9 editing using a single sgRNA. (Q Wang et al., 2018) According to recent research, CRISPR/Cas9 has the ability to cleave non-integrated HIV-1, which would lead to a 3–4-fold decrease in integrated HIV-1 provirus. Surprisingly, the non-integrated HIV-1 provirus responds to the DNA repair process mediated by non-homologous end joining (NHEJ). (Mefferd et al., 2018) Consequently, CRISPR/Cas9 exhibits efficacy in dormant cells, acting on both HIV-1 and proviral DNA, thereby enhancing its potential and promise for the treatment of HIV/AIDS. (Xiao et al., 2019)

The exploration of potential treatment strategies for HIV infection, focusing on the editing of the C—C chemokine receptor type 5 (CCR5) gene, was initiated following the discovery that CCR5 serves as an R5-HIV-1 co-receptor, providing protection against HIV infection in individuals lacking CCR5. Cho et al. used the CRISPR/Cas technology to specifically cut the viral DNA in human cells for genome editing. The results illustrated the efficient cleavage of CCR5 at the anticipated site, resulting in the generation of insertions or deletions (InDels) with a frequency exceeding 11 %. (Cho et al., 2013)

### 3.2 Hepatitis C virus (HCV)

Chronic infections with the HCV RNA virus constitute the primary etiology of hepatocellular carcinoma (HCC), liver cirrhosis, and liver fibrosis. (Bakhrebah et al., 2018) It has been found that the CRISPR-FnCas9 can be employed for preventing HCV infection in eukaryotic cells. (Sharma et al., 2021) A subgenomic viral replicon (JFH1/SG-Feo) containing the firefly luciferase protein fused in frame with neomycin phosphotransferase II (nptII) and five HCV nonstructural (NS) proteins was utilized to assess the suppressive activities of Cas13a on HCV RNA replication by Saeed et al. (Saeed et al., 2012) Although the encephalomyocarditis virus (EMCV) internal ribosome entry site (IRES) controlled the NS3-NS5B proteins, HCV IRES directed the expression of firefly luciferase. The Cas13a dramatically decreased the viral RNA levels in all IRES-targeting crRNAs following transiently transfecting Huh 7.5 cells with in vitro produced JFH1/SG-Feo RNA and then introducing the pCas13a-gRNA plasmid. At 48 h post-transfection, the knockdown efficacy of Leptotrichia shahii (Lsh) Cas13a resulted in a reduction of HCV RNA levels in cells, ranging from 3.5 to 8-fold. These findings imply that Cas13a-mediated targeting of the HCV IRES is extremely effective. In addition, the presence of Cas13a-crRNA plasmids that target the HCV IRES effectively reduced the translation of HCV RNA levels. (Ashraf et al., 2021)

### 3.3 Influenza virus

Annually, seasonal influenza virus epidemics claim the lives of 290,000 to 650,000 individuals worldwide, while significant pandemics causing substantial human morbidity and mortality occurred in 1918, 1957, 1968, and 2009. (Neumann et al., 2009; Basler and Aguilar, 2008) Influenza A virus (IAV), which belongs to the family Orthomyxoviridae, is a negative sense ssRNA virus affecting both avian and mammalian species severely. The IAVs genome comprises eight viral RNA segments, and these segments each code for at least 11 proteins. IAV constantly develops new strains as a result of its propensity to acquire mutations; this necessitates the development of novel vaccinations and/or antiviral techniques. (Petrova and Russell, 2018) Additionally, waterfowl and wild birds always harbor highly dangerous avian influenza viruses, including H5 and H7 viruses, which occasionally transcend species barriers to endanger human health. (Li et al., 2014; Neumann et al., 2010) Since 2003 In over 60 countries on three continents, H5N1 viruses have spread the viral infection to both wild and domesticated birds. (Reed et al., 2014)

An experiment on a DF1 cell line stably expressing Cas13a, sequence-specific targeting of RNA by crRNAs and Cas13a has shown promising results for the control of infection. ^64^ This suggests that Cas13a can be utilized as a substitute to the currently used siRNA knockdown techniques to down-regulate the endogenous mRNA for functional research. The great degree of accuracy that Cas13a offers while permitting potent mRNA degradation is one of the obvious advantages of utilizing it instead of siRNAs. (A Challagulla et al., 2021) The information gathered lays the groundwork for testing Cas13a's antiviral activity against strains of highly pathogenic avian influenza like H5N1 or other viruses that affect poultry. By using techniques like the Tol2 transposon, it is possible to successfully insert anti-viral CRISPR/Cas13a transgenes into the chicken genome to produce transgenic chickens’ germline that expresses Cas13a and crRNAs that target H5N1, and then an antiviral impact could be shown through in vivo challenge studies. (Challagulla et al., 2020; A Challagulla et al., 2021)

### 3.4 Severe acute respiratory syndrome coronavirus type 2 (SARS-CoV-2)

A positive-sense ssRNA enveloped virus,SARS-CoV-2, which is the primary cause of the COVID-19 pandemic, generally attacks respiratory systems, both upper and lower. (Bojkova et al., 2020) The SARS-CoV-2 has the largest known RNA that is almost 30 kb in size. It is noteworthy that the SARS-CoV-2 genome codes for four structural proteins and 16 non-structural (NSP) proteins (including spike (S), nucleocapsid (N), membrane (M), and envelope (E) proteins). (Davidson et al., 2020; Kandeel et al., 2020) The activity of structural and non-structural proteins is directly connected to the pathophysiology and pathogenicity of SARS-CoV-2. (Gorla and Rao, 2020) In order to interact to the host receptors, the SARS-CoV-2 spike glycoproteins are absolutely necessary. The S proteins bind to angiotensin-converting enzyme 2 (ACE2), which acts as the virus's point of entry into the host cell. When the virus enters the host cells, the endoplasm reticulum (ER) causes these proteins to turn into highly N-glycosylated, which enables the virus to circulate without being recognized by the host immune system. (Nunes-Santos et al., 2021) SARS-CoV-2 is the cause of both pneumonia and acute respiratory distress syndrome (ARDS), and their pathogenesis is highly complicated. (Pedersen and Ho, 2020)

The CRISPR/Cas13d system has recently been employed to target RNA molecules, with Nguyen et al. suggesting its application to generate crRNAs specifically directed towards the viral S and ORF1ab (replicase-transcriptase) genes for the precise degradation of the SARS-CoV-2 RNA genome. (Nguyen et al., 2020) Owing to the Cas13d's small size (967 amino acids), high specificity, and excellent catalytic activity in mammalian cells for targeting the SARS-CoV-2 genome, it was chosen and delivered via "all in-one" AAV delivery vector with a sgRNA array. (TR Abbott et al., 2020) There are a number of factors that make CRISPR/Cas13d an effective COVID-19 treatment option for affected individuals. Given that it causes loss-of-function phenotypes without genetic loss of the targeted gene, the identified Cas13 effectors are capable of efficiently cleaving complementary target ssRNA, making them a more dependable and appropriate substitute for Cas9. As Cas13 does not specifically require PAM-like (i.e., NGG) at the editing site, the CRISPR/Cas13d system is versatile in constructing guide RNA using any sequence found in the genome of the virus. Therefore, it satisfies the requirement for the quick generation of gRNAs to target various viral strains that change and may resist conventional treatments. (Ali et al., 2018) Recent research by Zeng et al. has shown that CRISPR/Cas13d gives a broad-spectrum antiviral that can block a variety of human coronavirus strains and SARS-CoV-2 variants with more than 99 percent reduction in viral titer. (Zeng et al., 2022) They demonstrated that the spatial colocalization of the target viral RNA and crRNA within the cell is essential for Cas13d-mediated coronavirus suppression. Cas13d can considerably improve the therapeutic benefits of several small molecule medications against coronaviruses for either prevention or therapy, including agents targeting viral entry, viral RNA synthesis, and drugs having synergistic effect in combination with remdesivir and the most effective combination decreased viral titer by almost four orders of magnitude. Zeng et al. showed that the lipid nanoparticle delivery of Cas13d can successfully inhibit Omicron SARS-CoV-2 infection in human primary airway epithelium air-liquid interface cells. (Zeng et al., 2022)

Comprehension of the virus–host cell interactions necessary for successful replication of the virus appears to depend on the identification of host dependency factors, which can also serve as a framework for novel therapeutic approaches to treat COVID-19 infection and other coronaviruses that could surface later. In this regard, Kratzel et al. conducted two separate genome-wide loss-of-function CRISPR screens using human coronavirus-229E, an endemic coronavirus that causes mild symptoms of respiratory infection in humans, and MERS-CoV, a highly pathogenic coronavirus, to discover major host dependence factors crucial for coronavirus infection. (Kratzel et al., 2021) In both coronavirus screens, they discovered that a number of genes involved in autophagy, including membrane integral NOTCH2 Associated Receptor 1 (MINAR1), FK506 binding protein 8 (FKBP8), vacuole membrane protein 1 (VMP1), and transmembrane protein 41B (TMEM41B), were among the top hits. This suggests that host contributing factors in autophagy are necessary for the replication of coronavirus and can be considered as novel therapeutic targets. (Kratzel et al., 2021) Table 2 shows preclinical studies on CRISPR/Cas for the treatment of RNA virus infections.

**Table 2:** CRISPR/Cas research for the treatment of different RNA virus infections.

Type of infection	Disease-Related Gene/Protein	Results	References
HIV	LTRs j	Induction of mutation in LTRs of HIV-1^e^ DNA.	. (H Ebina et al., 2013)
HIV	CCR5 ^a^	Artificial chimeric RNAs and the Cas9 protein cleaved the CCR5 in the predicted location, causing up to 11 % of InDels to be induced.	. (Cho et al., 2013)
HIV	LTR and structural regions	Interference with the integrated proviral DNA in infected cells, which prevents the spread of new viruses.	. (Q Wang et al., 2018)
HIV	Tat, TAR m	Reduced the integrated HIV-1 provirus by three to four times by cleaving the non-integrated HIV-1 via NHEJ-mediated DNA repair.	. (Mefferd et al., 2018)
HIV	CXCR4 c	Resistance of the modified cells to X4 type HIV-1 infection.	. (Hou et al., 2015)
HIV	Gag, env, pol, vif, rev, LTR	Disruption of integrated lentivirus, decreased the GFP and p24expression, induced immunization against new infection , increased cell viability and had no off-target cleavage.	. (H-K Liao et al., 2015)
HIV	LTR	Decreased the viral DNA and RNA load, decreased the expression of p24.	. (Bella et al., 2018)
HIV	CXCR4-P191A	Reduced the p24 expression, luciferase expression, viral RNA burden, and host resistance to X4-trophic HIV-1 infection with no off-target alterations and no influence on the survival of the cells.	. (S Liu et al., 2018)
HIV	miR-146	Reduced the amount of viral RNA, p24, and GFP expression by downregulating SAHA; the host did not exhibit any off-target mutations; and the amount of NF-kB and NF-kB-regulated cytokines, including Type I IFN g, ISG i, PD-1^l^, and CTLA-4 b, increased.	. (Teng et al., 2019)
HIV	CXCR4 and CCR5	Reduced the p24 expression, resistance to HIV-1 infection that is X4-trophic and R5-trophic, no differences in apoptosis, no impact on cell survival, and no off-target alterations.	. (Yu et al., 2018)
HIV	TRIM5( αR332G and R355G	Showed no restriction on HIV-1 activity and induced undesired mutations.	. (Dufour et al., 2018)
HCV	JFH1/SG-Feo	Inhibition of Cas13a on HCV RNA replication.	. (Saeed et al., 2012)
HCV	HCV IRES	HCV RNA levels were 3.5–8 times lower as a result of the knockdown action.	. (Ashraf et al., 2021)
HCV	HCV UTR	Inhibition of virus replication and 40 % reduction in the translation of HCV proteins.	. (AA Price et al., 2015)
HCV	5′UTR, 3′UTR	Reduced the expression of E2, inhibition of HCV luciferase production, viral translation and replication.	. (AA Price et al., 2015)
Influenza	PB1, NP, and M	Viral titer levels in infected cells decreased as a result of PB1, NP, and M gene knockdown.	. (A Challagulla et al., 2021)
Influenza	NA	Inhibition of viral replication in human lung epithelial cells.	. (TR Abbott et al., 2020)
Influenza	IRF7 h gene	knock down the IRF7 gene, increased the amount of infected HEK293FT cells at late stages of infection and inhibited the development of viral infection.	. (Komissarov et al., 2019)
Influenza	IFIT2 f	IFIT2 knockout promoted the translation of viral mRNAs by suppressing ribosome pausing.	. (Tran et al., 2020)
Influenza	METTL3	Disruption of METTL3, decreased viral structural protein levels, virus titer and viral mRNA levels.	. (Winkler et al., 2019)
SARS-CoV-2	N1	Degradation of N1	. (Safari et al., 2021)
SARS-CoV-2	S protein and ACE2	Inhibition of the interaction between the S protein of SARS-CoV-2 and ACE2 on host cells, which reduces the amount of viral infectivity and inhibits viral replication.	. (Nalawansha and Samarasinghe, 2020)
SARS-CoV-2	FKBP8 d, TMEM41B n, VMP1 p, MINAR1 k	Targeted the autophagy-related genes and decreased the viral replication.	. (Kratzel et al., 2021)

^a^CCR5; chemokine receptor type 5.

b CTLA-4; cytotoxic T-lymphocyte-associated protein 4.

c CXCR4; C-X-C chemokine receptor type 4.

d FKBP8; FK506 binding protein 8.

e HIV-1; human immunodeficiency virus-1.

f IFIT2; interferon-induced protein with tetratricopeptide repeats 2.

g IFN; interferon.

h IRF7; interferon-regulatory factor 7.

i ISG; interferon-stimulated gene.

j LTRs; long terminal repeats.

k MINAR1; membrane integral NOTCH2 associated receptor 1.

l PD-1; programmed death-1.

m TAR; trans-activation response.

n TMEM41B; transmembrane protein 41B.

o TRIM5 α; tripartite motif-containing protein 5 alpha.

p VMP1; vacuole membrane protein 1.

## 4 Novel treatment strategies using CRISPR technology for SARS-CoV-2

### 4.1 AntiBody and Cas fusion (ABACAS)

In this technique, Cas13 is able to attach to one of the main proteins on the surface of the virus, such as the S protein which is the primary protein involved in mediating viral infection. (Thakore et al., 2015) Given that ACE2 (Angiotensin-Converting Enzyme 2) is the SARS-CoV-2 receptor to bind and enter human cells, a human ACE2 promotor can be used to regulate the expression of Cas13d that is inserted in its downstream in lung ACE2-positive cells. (Lan et al., 2020) The Cas13 nuclease can be fused to an antibody fragment that is unique to the S protein of SARS-CoV-2 to attach the CRISPR components. The ABACAS fragment specific to the S protein of SARS-CoV-2 is the name of the suggested method. (Thakore et al., 2015) To deliver CRISPR components to infected cells specifically, ABACAS technology uses the viral S protein. Even before the viral RNA translates into RNA-dependent RNA polymerase (RdRp) and replicates its genome, CRISPR components may easily access it. In addition to the selective delivery of CRISPR components, antibody fragments can act as a neutralizing agent, and by interfering between the S protein and ACE2 receptor reduce viral entrance into host cells. Hence, by preventing the S protein of the SARS-CoV-2 from interacting with ACE2 on the host cells, ABACAS may offer a dual strategy by decreasing viral replication and reducing viral infectivity. These segments of antibodies that are particular to the ACE2-binding S protein domain would have a "dual" impact when used in ABACAS design. Firstly, it acts as a prophylactic agent by preventing SARS-CoV-2 from binding to its receptor ACE2, and secondly, ABACAS can cleave the viral RNA within the host cell. (Nalawansha and Samarasinghe, 2020)

### 4.2 The peptidase domain of ACE2 and Cas13 (PDCas13) fusion

In the case of the generation of ABACAS-induced treatment-resistant mutations, a different strategy to accomplish selective and effective delivery of CRISPR components might be proposed. The ACE2 peptidase domain (PDCas13 fusion) or a short ACE2 peptide that was found to attach to SARS-CoV-2 through S protein can be used as an alternative to ABACAS. (Zhang et al., 2020). Recombinant human ACE2 protein (rhACE2), which interacts with ACE2 receptor, possesses the capacity to competitively block the host cell's ACE2 receptor's interaction with S protein when it is supplied in a soluble form. As a result, rhACE2 masks the presence of SARS-CoV-2. (Pang et al., 2020)

The PD domain of human ACE2 is recognized for its interaction with the receptor-binding domain (RBD) of the S protein. Consequently, PDCas13 interact with SARS-CoV-2 through the S protein. As a result, it would make the transport of CRISPR components into infected cells more effectively, and since the rhACE2 may have neutralizing effects as well, it may potentially have preventive effects. Even if rhACE and Cas13 fuse, it would still facilitate Cas13′s attachment to the viral particle via S protein despite the limited neutralizing activity. (C Lei et al., 2020) Indeed, PDCas13 would infiltrate virus-infected cells and, once internalized, would initiate an antiviral reaction. (C Lei et al., 2020)

### 4.3 Prophylactic antiviral CRISPR in huMAN cells (PAC-MAN)

It has been demonstrated that the SARS-CoV-2 genetic material can be cleaved utilizing Cas13d and crRNA. (TR Abbott et al., 2020) The innovative technology behind PAC-MAN strategy was invented by Abbott et al. In this strategy, Cas13d was used as an endonuclease. They designed numerous crRNAs that can target various coronaviruses by using a bioinformatic process to determine areas that are conserved in SARS-CoV-2. This technique involves Cas13d recruiting crRNAs with a programmable 22-nucleotide spacer sequence. To destroy these conserved areas, the Cas13d protein is directed to certain RNA targets by this spacer region. Due to Cas13d's superior on-targeting capacity on mammalian cells, this nuclease is used to target viruses such as SARS-CoV-2. (Yan et al., 2018) It is important to mention that a pool of crRNA could inhibit the reporter signal by around 70 %, demonstrating the ability of CRISPR PAC-MAN technology to eliminate viral genetic material. Furthermore, several crRNAs that target the entire conserved region of the RdRP and the SARS-CoV-2 virus's N protein genes led to higher than 80 % and 90 % of the RNA to degrade, respectively. Therefore, SARS-CoV-2, IAV, and perhaps all strains of sequenced coronaviruses can be the targets of the PAC-MAN approach. (TR Abbott et al., 2020)

The ability of PAC-MAN to tackle several coronaviruses with a single cocktail of various crRNAs that target conserved areas in various coronaviruses is one of its main advantages. Surprisingly, a computational study forecasts that three crRNAs are sufficient to target all beta coronaviruses linked to SARS, MERS, and COVID-19. Furthermore, both DNA and (±) RNA viruses can be targeted by PAC-MAN technology and the evolution of mutations can be readily combated given the option of focusing on conserved sequences and the potential of utilizing numerous crRNAs. With relatively small systemic alterations, this technique may be quickly implemented at the very beginning of the next pandemic once the safety of CRISPR technology in humans is established. (Lotfi and Rezaei, 2020) Fig. 2 represents a schematic for the aforementioned three novel treatment strategies using CRISPR/Cas technology for COVI-19 treatment.

**Fig. 2:**
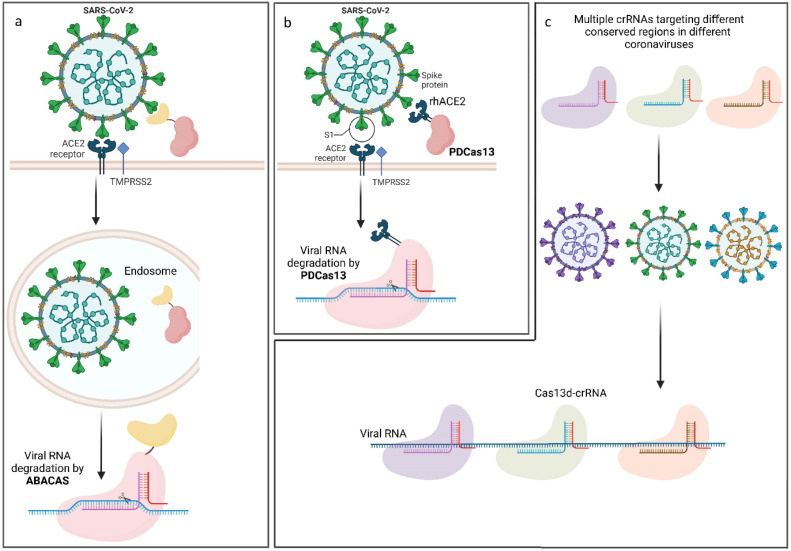
Novel treatment strategies using CRISPR technology for the treatment of SARS-CoV-2. a) AntiBody And CAS fusion (ABACAS) strategy b) Fusion Biologics Comprising the Peptidase Domain of ACE2 and Cas13 (PDCas13) c) Prophylactic Antiviral CRISPR in huMAN cells (PAC-MAN) strategy.

## 5 CRISPR/Cas technology in human vaccine development

Undoubtedly, vaccination has been a key factor for battling against several infectious diseases that infect both humans and animals. Many fatal diseases, like smallpox and rinderpest, have been eradicated worldwide as a result of the broad global use of efficient vaccinations. The introduction of recombinant vaccines has enabled CRISPR/Cas9-based genome editing that is one of the most effective, adaptable, and flexible techniques for gene editing, as a potent strategy for the development of these vaccines. The optimal vaccines with a great level of immune system response potential are live attenuated vaccines. The complexity and the high cost of manufacturing these viruses are the key obstacles to their development. These live attenuated vaccines can be obtained at an inexpensive and large-scale production by using the CRISPR toolkit to randomly modify the viral genome and choosing a live attenuated virus tailored to non-permissive cells. Additionally, this method can be utilized to investigate viral evolution and forecast potential virus alterations in the upcoming years. (Jamehdor et al., 2022) Generation of live attenuated vaccines and recombinant viral vector vaccines for human diseases using CRISPR/Cas technology have been developed for some viral infections in recent years.

Sporadic transfer of highly pathogenic avian influenza viruses (AIVs) from poultry to humans increases the constant threat of a pandemic. Instead of eradicating AIVs, the current immunization efforts focus on preventing avian infection using vaccines. (Vilela et al., 2020) Birds remain vulnerable to influenza A viruses, notwithstanding the present efficacies of various immunization techniques. CRISPR/Cas9 has been employed to generate viral-based vaccines that protect against particular avian influenza virus subtypes. Research has demonstrated the potential of CRISPR/Cas9 technology to target the Duck Enteritis Virus (DEV) genome and produce a vaccine candidate that expressed the hemagglutinin (HA) protein of the highly deadly avian influenza virus H5N1. (Zou et al., 2017) The recombinant DEV vaccine was designed to include the pre-membrane proteins and envelope glycoprotein genes of the duck tembusu virus (DTMUV), thereby establishing a trivalent vaccination candidate effective against H5N1, DEV, and DTMUV infections. (Zou et al., 2017) Chang et al. investigated homology-directed repair (HDR)-dependent CRISPR/Cas9 as a tool for generating Turkey herpesvirus (HVT)-Avian influenza viruses (AIVs) bivalent vaccines. (Chang et al., 2019) The HVT intergenic region, which is located between UL45 and UL46, was modified to accept a H7N9 HA expression cassette which is nonpathogenic virus of domestic turkeys. They quickly created recombinant HVT-H7HA candidate vaccines using CRISPR/Cas9 and used erythrocyte binding to maximize the selection efficiency of the bivalent vaccine. Erythrocyte-based screening is less expensive, faster, and applicable to recombinant viruses that express the majority of influenza virus's HA, except for a few recent human influenza viruses whose HA lost its capacity to bind to erythrocytes. Any target antigen that exhibits erythrocyte adsorption activity is suitable for the erythrocyte-adsorption test. Therefore, erythrocyte selection combined with HDR-CRISPR/Cas9 is a potent and fast approach for creating recombinant avian influenza vaccines. (Chang et al., 2019)

For the purpose of developing animal and cell models for vaccine development research, CRISPR/Cas technology can also be utilized. Ensuring the preservation of the glycosylation profile and antigenic characteristics of the influenza virus, which are pivotal for influenza monitoring and vaccine development, is essential during its propagation in human-origin cell cultures. However, only few cell lines, none of which are human-derived, are extremely tolerant to the influenza virus. The barrier may be related to host restriction factors that prevent influenza development, such as the protein AnxA6 that inhibits the packing of influenza virion. The CRISPR/Cas9 mediated deletion of the ANXA6 gene was investigated by Komissarov et al., as an approach to get beyond the host restriction barrier and make human cell lines more vulnerable to influenza infection. (Komissarov et al., 2022)

To effectively prevent pandemics such as the previously COVID19 pandemic, a universal platform that can quickly produce multiplex vaccine candidates is essential. Zhu and colleagues have established a platform by employing CRISPR to modify the bacteriophage T4. (Zhu et al., 2021) By using the CRISPR technique they inserted different viral components into the proper compartments of phage nanoparticle structure to develop a novel vaccine candidate. These comprise expressible spike genes in the genome, surface-decorated spike and envelope epitopes, and packaged nucleocapsid proteins. In animal models, phage displaying with trimers spike protein was determined to be the potent vaccine candidate. This vaccine induced strong immune responses, both T helper 1 and T helper 2 IgG subclasses, prevented the interactions between virus and receptor, neutralized viral infection, and provided complete protection against viral challenge without the need for an adjuvant. (Zhu et al., 2021) Therefore, the deployment of efficient phage-based vaccinations against any infectious diseases can be possible with this novel nanovaccine design platform.

## 6 Clinical trials

The prospect of employing CRISPR as an antiviral technique offers promise for potential therapeutic applications in clinical settings. Hence, CRISPR technology has advanced to clinical trials for the recent treatment of various viral diseases. Ex vivo ablation of the HIV co-receptor CCR5 from CD34^+^ hematopoietic progenitor cells was the goal of the first planned clinical study utilizing anti-HIV CRISPR/Cas9 (NCT03164135). The goal of the first CRISPR/Cas9 clinical experiment to specifically target human viral DNA was to damage HPV E6/E7 DNA in HPV-related cervical intraepithelial neoplasia (NCT03057912). The CRISPR/Cas9-based gene editing clinical trials that are currently being conducted to treat viral infections are listed in Table 3.

**Table 3:** Currently registered clinical trials with CRISPR/Cas-Based gene editing for the treatment of viral infections.

NCT Number	Disease	Target Gene/Effect	Phase	Country
NCT03057912	Human Papillomavirus-Related Malignant Neoplasm	E6 and E7	I	China
NCT03164135	HIV	CCR5 b	Not specified	China
NCT05144386	HIV-1-infection	HIV replication system	I	United States
NCT04560790	HSV-1- induced viral keratitis, blindness eye, herpes simplex virus infection, and cornea	HSV-1 viral gene	I/II	China
NCT03044743	EBV-positive, advanced-stage malignancies	PD-1 c	I/II	China
NCT04990557	COVID-19	PDCD1 d and ACE2 a	I/II	Not Provided

a ACE2; angiotensin converting enzyme-2.

b CCR5; chemokine receptor type 5.

c PD-1; programmed death-1.

d PDCD1; Programmed cell death protein 1.

## 7 Challenges and potential solutions

While the CRISPR/Cas technology presents several advantages, it is not without limitations. The absence of sufficient preclinical and clinical evidence showcasing the technology's suitability for human use necessitates additional clinical trials before its deployment becomes viable during the current pandemic. The success rate of CRISPR/Cas9-mediated gene editing in humans is mostly influenced by the following parameters.

### 7.1 Off-target effects

Since off-target effects are mostly controlled by sgRNAs, rational sgRNA design is required to ensure the effectiveness of CRISPR/Cas9 gene editing methods. Moreover, the application of the CRISPR/Cas9 system in human contexts can achieve precise regulation of its off-target performance by employing an alternative Cas9 variant, such as Cas9 nickase. This variant, characterized by reduced off-target properties, is activated through single-stranded DNA cleavage as opposed to double-stranded DNA cutting. (Ran et al., 2013)

Another strategy proposed the use of an inert fusion protein made of Cas9 and the FokI endonuclease, which only becomes active after accurate sgRNA binding to both forward and reverse DNA sequences. This method inhibits cleavage at off-target locations by inducing single-stranded cleavage. (Tsai et al., 2014) In addition, anti-CRISPR proteins, naturally occurring inhibitors of CRISPR/Cas complexes, are another strategy that can be used to decrease off-target cleavages. (Shin et al., 2017)

### 7.2 The need to recognize PAM sequence

Before activation, Cas12 and Cas9 must also detect PAM sequences, which may restrict targeting and influence the effectiveness and flexibility of editing . (Marraffini and Sontheimer, 2010) In this regard, utilizing almost PAM-less engineered Cas enzymes can virtually eradicate PAM's drawbacks . (Ding et al., 2021; Zhao et al., 2023)

### 7.3 Efficient and targeted delivery of the CRISPR/Cas cargo

The specific and targeted delivery of CISPR/Cas cargo is a crucial step for its efficient application and reducing the risk of off-target effects. Hence, before testing CRISPR antiviral therapies in the clinic, a successful delivery method must be designed. (Nalawansha and Samarasinghe, 2020) As a result, more effort needs to go into choosing the best delivery method based on the size, charge, and the composition of the CRISPR/Cas9 cargo. For the delivery of CRISPR/Cas9 many physical, viral, and non-viral approaches have been employed. Viral vectors are widely used for the delivery of CRISPR/Cas9 in both in vitro and in vivo settings. However, they face a number of obstacles, including size restrictions to carry foreign genes and plasmids, post-integration of viral genome into the host genome and development of cancer, challenges in mass manufacturing, and robust immune responses. Therefore, by using non-viral vectors such as polymer or lipid nanocarriers as an alternative delivery strategies, these restrictions may be tackled. (L Li et al., 2018)

### 7.4 Immunogenicity and toxicity of Cas proteins

Due to the prokaryotic origin of Cas proteins, their in vivo administration can potentially induce toxicity in human cells, along with an immunological response leading to the production of antibodies specific to Cas proteins within the host body. The Cas9 protein can undergo targeted mutations to eliminate immunodominant epitopes, thereby maintaining its function and specificity. In this regard, Ferdosi et al., demonstrated that two mutations in the cas9 protein's epitope binding residues lessen the protein's immunogenicity while preserve its specificity and activity. (Ferdosi et al., 2019) This approach becomes particularly crucial when there is a need for prolonged expression of Cas9 over an extended period. Nevertheless, further investigations are imperative to mitigate the limitations associated with this technology and enhance its overall efficacy and safety.

## 8 Conclusion

The CRISPR system has emerged as an innovative technological tool that contributes to the development of novel, highly targeted antiviral treatments and vaccines. The rapid advancement and significant strides in the realm of CRISPR/Cas9 have encompassed a broad spectrum, transforming it into a significant domain within biotechnology. It has facilitated fundamental research, offering diverse applications in genetic engineering. Despite the substantial knowledge gained, numerous pivotal questions in this field remain unresolved. Hence, ongoing in-depth investigations and rigorous clinical validation efforts are pivotal components in realizing its prophesied impact. Nevertheless, the routine clinical application of CRISPR technology encounters substantial obstacles that necessitate concerted efforts for resolution. Addressing these challenges is paramount before the technology can seamlessly integrate into clinical settings. A forward-looking perspective advocates for a dedicated focus on further research endeavors, aiming to comprehensively assess the efficacy and safety of CRISPR treatment protocols. As we navigate the dynamic landscape of viral therapeutics, the judicious and strategic deployment of CRISPR promises not only to mitigate current challenges but also to chart a transformative course in the proactive management of infectious diseases on a global scale. Looking further ahead, the future development of CRISPR technology holds exciting possibilities. Advances in off-target effects mitigation, enhanced delivery systems, and the exploration of CRISPR beyond antiviral applications into areas like genetic disorders and cancer therapy are on the horizon. The convergence of CRISPR with other cutting-edge technologies, such as nanotechnology, opens new frontiers for synergistic interventions. By embracing this forward-looking perspective, we not only fortify our current arsenal against infectious diseases but also sow the seeds for a future where CRISPR becomes a transformative force in the broader landscape of precision medicine and therapeutic innovation.

## CRediT authorship contribution statement

**Farzaneh Zahedipour:** Writing – original draft. **Fatemeh Zahedipour:** Writing – review & editing. **Parvin Zamani:** Writing – review & editing. **Mahmoud Reza Jaafari:** Writing – review & editing, Conceptualization. **Amirhossein Sahebkar:** Writing – review & editing, Conceptualization.

## Declaration of competing interest

The authors declare that they have no known competing financial interests or personal relationships that could have appeared to influence the work reported in this paper.

## Data Availability

No data was used for the research described in the article.
